# Characterization of non-tuberculous mycobacterial pulmonary disease in Nanjing district of China

**DOI:** 10.1186/s12879-019-4412-6

**Published:** 2019-09-02

**Authors:** Chunmei Hu, Lili Huang, Min Cai, Weixiao Wang, Xudong Shi, Wei Chen

**Affiliations:** 1 Department of Tuberculosis, the second hospital of Nanjing, Nanjing University of Chinese Medicine, Nanjing, 210003 China; 2 Department of Science and Education, the second hospital of Nanjing, Nanjing University of Chinese Medicine, Nanjing, 210003 China; 3 Clinical Research Center, the second hospital of Nanjing, Nanjing University of Chinese Medicine, Zhongfu Road 1, Gulou District, Nanjing, 210003 Jiangsu China; 4 Clinical Laboratory, the second hospital of Nanjing, Nanjing University of Chinese Medicine, Nanjing, 210003 China

**Keywords:** Nontuberculous mycobacteria, Prevalence, Pulmonary disease, Clinical symptom, Comorbidity

## Abstract

**Background:**

Environmental bacteria, nontuberculous mycobacteria (NTM), are recognized as one of the major human infection pathogens. NTM are prone to be mistaken as multidrug-resistant *Mycobacterium tuberculosis* and challenge our fight against TB. In addition, treatment of NTM per se is intractable. Remarkably, the distribution of NTM pathogenic species is geographically specific. Thus, it is very important to summarize the prevalent features and clinical symptoms of NTM pulmonary disease. However, In Nanjing district, southeast China, there is no such a report.

**Methods:**

Through investigating electronic medical records and analyzing data of clinical examination system (Lis), we retrospectively summarized the NTM species from 6012 clinical isolates from May 2017 to August 2018, and analyzed the association between NTM species and clinical symptoms.

**Results:**

Of 6012 clinical specimens, 1461 (24.3%) could grow in the MGIT 960 broth. Among these positive isolates, 1213 (83%) were *M. tuberculosis*, 22 (1.5%) were *M. bovis*, and 226 (15.5%) were NTM. After deducting redundancy, those NTM specimens were confirmed from 154 patients, among which, 87 (56.5%) patients met the full ATS/IDSA NTM disease criteria. The most common etiologic agent was *M. intracellulare* (70.1%). NTM infection was associated with age, based on which 68.6% male patients and 77.8% female patients were over 50 years old. The older patients were more likely to have hemoptysis, but the younger patients were more likely to manifest chest congestion. Male patients were more likely to have shortness of breath and females were more likely to have hemoptysis. The most common radiographic presentation of NTM pulmonary disease was bronchiectasis, accounting for 39.1%. Remarkably, multiple and thin-walled cavities were outstanding. The most frequent comorbidity of NTM disease was previous tuberculosis (64%), followed by clinical bronchiectasis (19.5%), HIV (19.5%), and 6.9% chronic obstructive pulmonary disease (COPD). There was no association between NTM species and clinical symptoms.

**Conclusion:**

This study retrospectively investigated the prevalence of NTM pulmonary disease in Nanjing district, southeast China. Similar to Beijing area, north China, *M. intracellulare* was the major pathogenic NTM species. Clinical symptoms of the disease were not species-specific. Previous TB and HIV infection immensely enhanced risk of NTM disease.

## Background

Nontuberculous mycobacteria (NTM) are a large group of naturally-occurring mycobacteria, ubiquitous in the environment like soil and water [1–3]. NTM are very diverse organisms, comprising over 170 species with different virulence features [4]. Some species are opportunistic pathogens for human beings, which could cause severe pulmonary and non-pulmonary diseases [2, 5, 6]. In the past, NTM infection did not draw much attention since it was not transmitted from person to person, and was often thought as lab contamination and neglected. However, in recent two decades, with more advanced diagnostic laboratory techniques and increased awareness among clinicians, global reports for incidence of NTM infection have dramatically increased [7–9]. NTM are already recognized as one of the major emerging pathogens [10]. Like MTB, NTM usually cause chronic lung infection. Indeed, it is difficult to distinguish the clinical symptoms between NTM disease and TB, and NTM is often misdiagnosed as multidrug-resistant MTB [11]. Treatment of NTM infection is also challenging, requiring multiple antimycobacterial drugs, expert management and longer course [12]. Therefore, it is imperative to summarize the clinical characteristics of NTM pulmonary disease, to facilitate diagnosis and treatment of NTM infection in time.

The distribution of NTM pathogenic species is geographically specific [13, 14]. *M. avium* complex is the major causative agent of NTM disease in Europe and North American [1]. In contrast, *M. abscessus* is most common in Asia and Oceania [13]. Even in Asia, the frequency of clinical isolation of NTM species is different between countries and cities. For example, *M. simiae* is the most frequently disease-causing NTM in Iran [15]. However, in South Korean, *M. avium* complex and *M. abscessus* are most commonly involved [16]. Thus, it’s necessary to investigate local prevalence of NTM infection.

China is one of the TB high burden developing countries. According to the WHO report on global tuberculosis in 2018, new infection of TB in China was estimated as 889,000 in 2017, ranking second in the world [17]. Incidence of NTM infection in China also rapidly increased in recent decades [18, 19]. So far, there are limited reports for characteristics of NTM pulmonary disease in China [20–23]. Especially, to our best knowledge, there is no evaluation of NTM prevalence in Nanjing, which is one of the most prosperous cities of Yangtze River Delta, southeast China. According to the report of Centers for Disease Control (CDC) of Nanjing in 2018, the annual incidence rate of TB is 26.43/100,000 (2203/8,335,000) in Nanjing. The second hospital of Nanjing is the unique designated hospital for diagnosis and treatment of infectious diseases in Nanjing district. In this retrospective study, we summarized the demographic and clinical features of patients with NTM lung disease in our hospital and analyzed the association between clinical symptoms and different NTM species.

## Methods

The retrospective study investigated and evaluated the patients with NTM pulmonary disease in Nanjing district of China from May 2017 to August 2018. A total of 6012 clinical specimens including sputum, endotracheal suction fluid, or alveolar lavage were processed following the previous protocol with sodium hydroxide–N-acetyl-l-cysteine and 0.7% chlorhexidine [24]. The pellets were cultured in the automated BACTEC MGIT 960 Mycobacteria culture system (BD), which usually reports positive results from two to four weeks and reports negative results after 42 days. According to the rules for Chinese clinical laboratory examination of tuberculosis [25], the positive mycobacterial growth was further cultured on two screening L-J medium, each containing either hydrazine thiophene-2-carboxylate (TCH) or p-nitrobenzoic acid (PNB). The strain that did not grow on both mediums was recorded as *M. bovis*. MTB could grow only on the former, and NTM could grow on both. All those mediums were purchased from Zhuhai Beisuo biotech company.

All the NTM species were identified using Mycobacterium Species Identification kit (PCR-reverse dot blot) of Da An Gene company [26, 27]. With this kit, 22 NTM species are routinely identified in clinical molecular detection laboratory. Briefly, species-specific fragments are amplified by PCR with primers labelled by biotin at 5′ end. Fragments are harvested and hybridized with the nylon membrane in the stringent hybrid condition, on which species-specific probes are immobilized. The coloration of different loci corresponds to different NTM species. With this commercial diagnostic kit, 22 NTM species could be identified, including: *M. smegmatis*, *M. intracellulare*, *M. kansasii*, *M. terrae*, *M. nonchromogenicum*, *M. tuberculosis* complex, *M. avium*, *M. scrofulaceum*, *M. xenopi*, *M. chelonae*, *M. gilvum*, *M. chelonae ss. Abscessus*, *M. phlei*, *M. fortuitum*, *M. porcinum*, *M. gordonae*, *M. triviale*, *M. gastri*, *M. vaccae*, *M. marinum*, *M. ulcerans*, *M. szulgai*, *M. diernhoferi*, *M. simiae*. It is important to note that the kit could not differentiate *M. chelonae ss. abscessus* from *M. chelonae*, *M. marinum* from *M. ulcerans, M. fortuitum* from *M. porcinum*. For convenience’s sake, we used *M. abscessus* to represent the complex of *M. chelonae ss. abscessus* and *M. chelonae.*

We collected and reviewed the electronic medical records of patients with NTM infection from medical system of our hospital. Evaluation of NTM lung disease was strictly based on the 2007 American Thoracic Society (ATS) and Infectious Disease Society of America (IDSA) guidelines [28], which briefly include the following: (1) pulmonary symptoms; (2) radiograph showing nodular or cavitary opacities; (3) at least one bronchial wash or lavage specimen positive, or repeated (two or more) sputum specimens positive. For each NTM patient, we analyzed their medical history, clinical symptoms, comorbidities, lab examination results and radiographic features. All data were analyzed by Chi-square test, Correction chi-square test, Fisher *P* values, unifactor and multifactor logistics stepwise regression analysis. *p* < 0.05 means statistical significance.

## Results

**Fig. 1:**
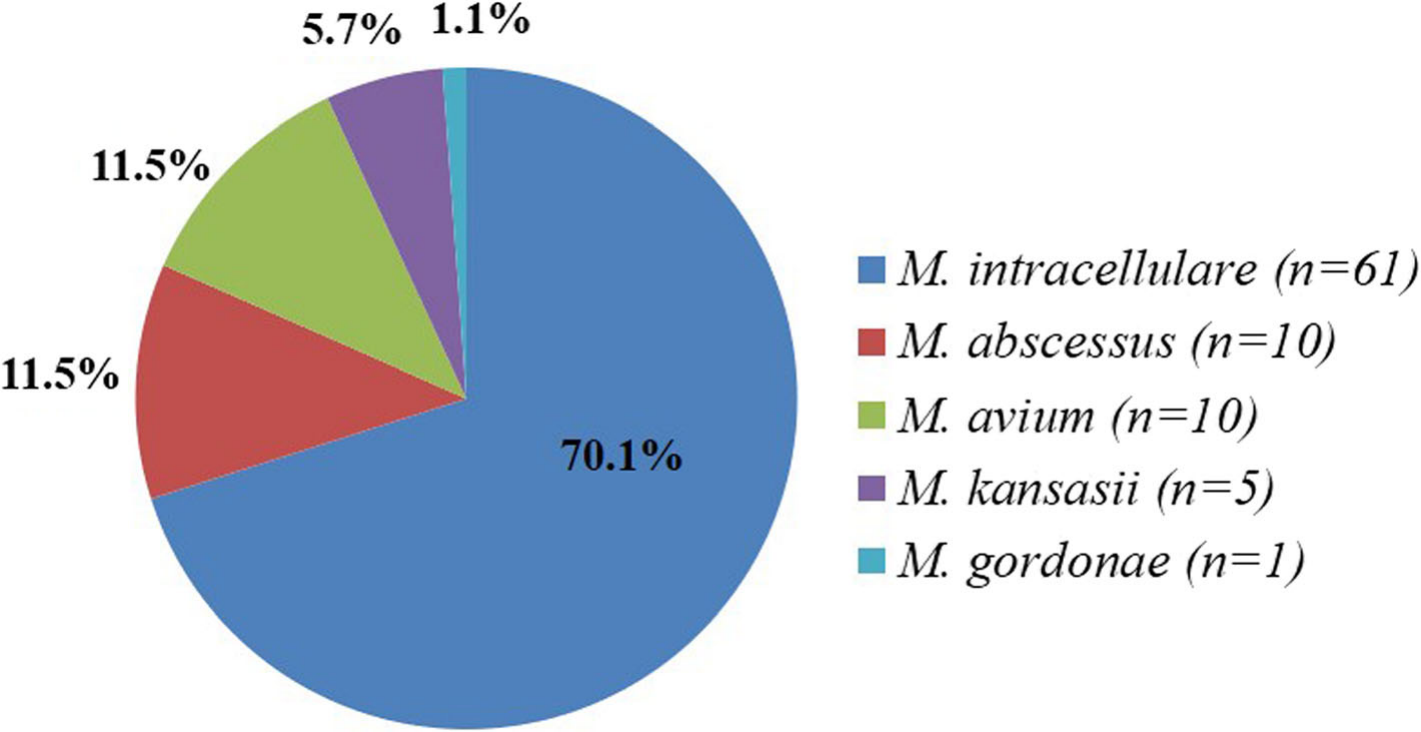
Distribution of etiologic agents of NTM lung disease in Nanjing district of China from May 2017 to August 2018 (*n* = 87). *M. abscessus* represented the complex of *M. chelonae ss. abscessus* and *M. chelonae*

A total of 6012 clinical specimens were cultured in the BACTEC MGIT 960 mycobacteria culture system and 1461 (24.3%) showed growth. Among these positive isolates, 1213 (83%) were MTB, 22 (1.5%) were *M. bovis*, and 226 (15.5%) were NTM. After deducting redundancy, those NTM specimens were confirmed from 154 patients. Considering culture results, radiographic features, medical records and clinical symptoms, 87 (56.5%) patients met the full ATS/IDSA NTM disease criteria and were diagnosed with NTM lung disease. The remaining 67 NTM cases (43.5%) did not meet all the criteria and were diagnosed as nonpathogenic colonization. The clinical records of 87 patients with NTM lung disease were available for review. Five different species of NTM were observed (Fig. 1). *M. intracellulare* was etiologic agent in most cases (*n* = 61, 70.1%). *M. abscessus* (representing the complex of *M. chelonae ss. abscessus* and *M. chelonae*, refer to Methods) and *M. avium* respectively accounted for 11.5% of isolates from NTM patients (*n* = 10). A small number of cases were due to *M. kansasii*, (*n* = 5, 7.5%) and *M. gordonae* (n = 1, 1.1%). Most pathogenic NTM in this study were slowly growing mycobacteria except for *M. abscessus*, which is a rapidly growing mycobacterium. Meanwhile, analysis of the erythrocyte sedimentation rates (ESR) of all NTM patients showed that 83.9% (73/87) of NTM disease patients had higher ESR, indicating chronic inflammation in those patients.

### Demographic feature of patients with NTM pulmonary disease

**Fig. 2:**
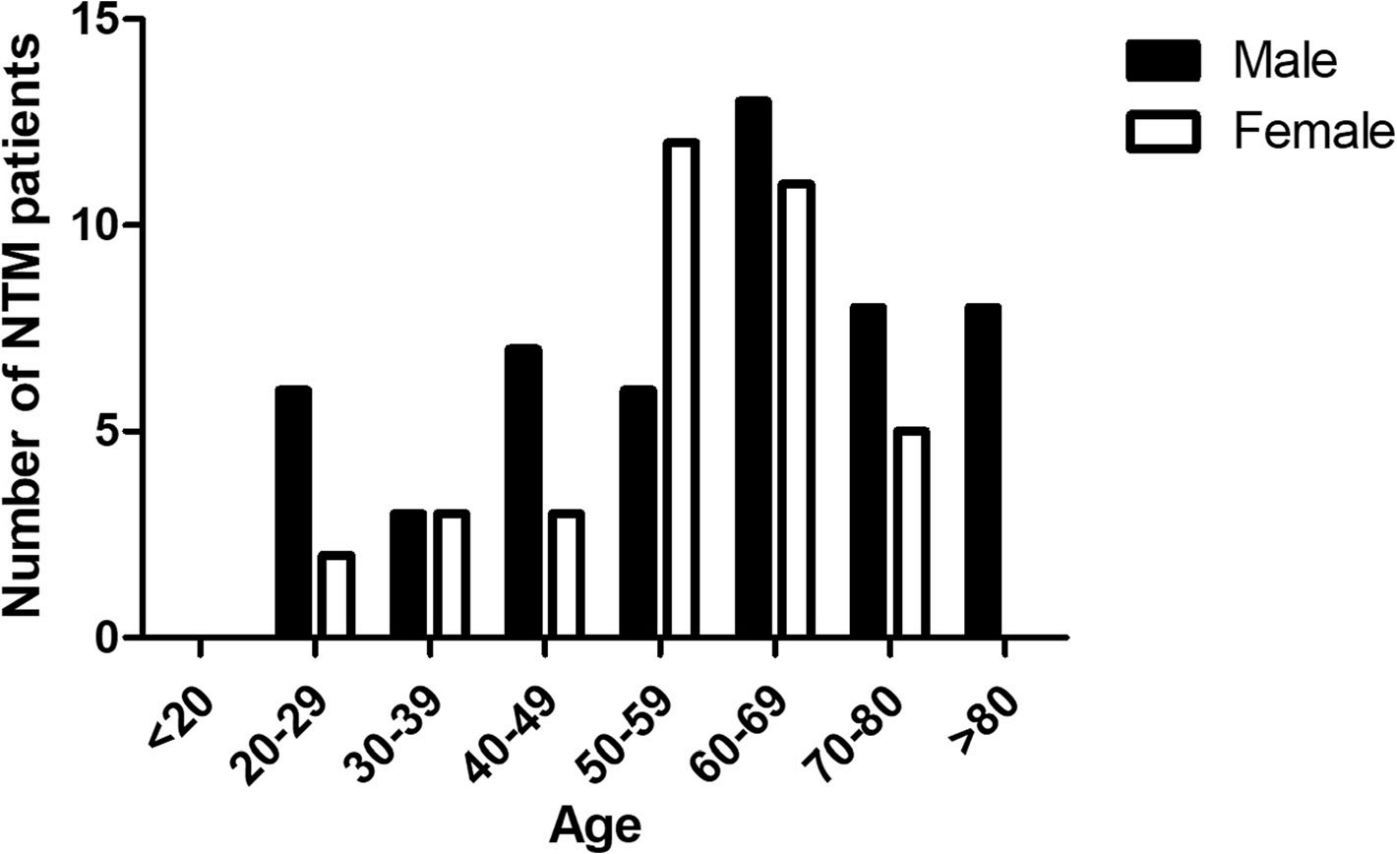
Demographic features of patients with NTM lung disease

The median age was 60 years for all 87 patients. Among them, 51 (58.6%) were male with median age 63 and 36 (41.4%) were female with median age 59 (Fig. 2). For all patients, 68.6% (35/51) male and 77.8% (28/36) female were over 50 years old, indicating that NTM infection was associated with age (Fig. 2). All five NTM species were found in male patients. In contrast, only *M. intracellulare* and *M. abscessus* were observed in the female patients.

### Radiographic characteristic of patients with NTM pulmonary disease

To determine whether different NTM species caused different pulmonary damage, we analyzed the radiographic characteristic of NTM patients. As shown in Table 1, the most common radiographic presentation of NTM pulmonary infection was bronchiectasis, accounting for 39.1% (34/87). Cavity and nodules were observed in more than one third patients. Remarkably, multiple and thin-walled cavities were outstanding. 18.3 and 8.0% NTM patients suffered from lymphadenovarix and pleural/pericardial effusion, respectively. After statistical analysis, there was no association between NTM species and radiographic characteristics.

**Table 1:** Radiographic characteristics of different NTM species infection

Presentations/No. of isolates	MAB (*n* = 10)	MAV (*n* = 10)	MKA (*n* = 5)	MIN (*n* = 61)	MGO (*n* = 1)	Total87	Percent
Bronchiectasis	7	2	4	20	1	34	39.1%
Pulmonary Cavity	6	2	2	23	0	33	37.9%
Single cavity	0	0	0	3	0	3	3.45%
Multiple cavities	6	2	2	20	0	30	34.48%
Thick-walled cavity	0	0	0	1	0	1	1.15%
Thin-walled cavity	6	2	2	22	0	32	36.78%
Nodule	5	4	2	22	0	33	37.9%
Lymphadenovarix	2	2	0	12	0	16	18.3%
Pleural/Pericardial Effusion	0	2	0	5	0	7	8.0%
Destroyed lungs	0	0	0	3	0	3	3.4%
Pneumothorax	0	0	1	1	0	2	2.3%
Total Percent	32 16.5%	16 8.2%	13 6.7%	132 68%	1 0.5%	194	

*MAB M. chelonae ss. abscessus* and *M. chelonae* complex, *MAV M. avium*, *MKA M. kansasii*, *MIN M. intracellulare*, *MGO M. gordonae*

### Clinical manifestation of NTM pulmonary disease

Presentation of NTM pulmonary infection included cough and expectoration, hemoptysis, chest congestion, shortness of breath, fever (as shown in Table 2). There was no association between clinical symptoms and NTM species. However, through Pearson chi square test, we did notice the association between shortness of breath and hemoptysis and gender. Male patients were more likely to have shortness of breath (χ2 = 0.255.41, *p* = 0.020), and females were more likely to have hemoptysis (χ2 = 4.74, *p* = 0.030). In addition, there was association between hemoptysis and chest congestion and age. The older patients were more likely to have hemoptysis (*r* = 0.26, *p* = 0.017), but the younger patients were more likely to manifest chest congestion (*r* = 0.24, *p* = 0.027).

**Table 2:** Clinical symptoms of NTM lung disease

Symptoms No. of isolates	MAB (*n* = 10)	MAV (*n* = 10)	MKA (*n* = 5)	MIN (*n* = 6)	MGO (*n* = 1)	Total87	Percent
Cough/Expectoration	8	9	5	45	1	68	78.2%
Hemoptysis	6	0	0	11	0	17	19.5%
Chest Congestion	1	0	0	7	0	8	9.2%
Shortness of breath	2	2	0	5	0	9	10.3%
Fever	1	5	3	15	0	24	27.6%
Total	18	16	8	83	1	126	
Percent	14.3%	12.7%	6.3%	65.9%	0.8%		

*MAB M. chelonae ss. abscessus* and *M. chelonae* complex, *MAV M. avium*, *MKA M. kansasii*, *MIN M. intracellulare*, *MGO M. gordonae*

### Comorbidities of NTM pulmonary disease

Of 87 NTM patients, there were 30 cases (34.5%) which had no associated complications. The most common comorbidity of NTM infection was previous TB (56/87) (Table 3), which showed that previous MTB infection significantly increased risk for further NTM infection. The second comorbidity was HIV infection. 17 NTM patients were HIV positive. Also, clinical bronchiectasis was associated with NTM infection (17/87). A small part of NTM patients meanwhile had COPD (6/87), hepatitis (5/87) and diabetes mellitus (4/87) (Table 3). Through multifactor regression analysis, comparing with female, male NTM patients were more likely to co-infect HIV (OR = 20.92, 95% CI (2.60, 168.12)) and COPD (OR = 10.46, 95% CI (1.23, 88.97)). Interestingly, among NTM species, *M. intracellulare* infection was more likely to combine with COPD (OR = 0.18, 95% CI (0.04, 0.97)) and bronchiectasis (OR = 0.18, 95% CI (0.04, 0.72)).

**Table 3:** Comorbidities of NTM lung disease

Comorbidities No. of isolates	MAB (*n* = 10)	MAV (*n* = 10)	MKA (*n* = 5)	MIN (*n* = 6)	MGO (*n* = 1)	Total87	Percent
Previous TB	8	6	0	41	1	56	64.4%
Clinical Bronchiectasis	6	0	0	11	0	17	19.5%
HIV positive	0	8	4	5	0	17	19.5%
COPD	0	0	1	5	0	6	6.9%
Hepatitis	2	0	0	3	0	5	5.7%
DM	0	0	0	4	0	4	4.6%
Hypertension	1	0	0	3	0	4	4.6%
Cancer	0	0	0	3	0	3	3.4%
AS	0	1	0	0	0	1	1.1%
Pulmonary fibrosis	1	0	0	3	0	4	4.6%
Total	18	15	5	78	1	117	
Percent	15.4%	12.8%	4.3%	66.7%	0.9%		

*MAB M. chelonae ss. abscessus* and *M. chelonae* complex, *MAV M. avium*, *MKA M. kansasii*, *MIN M. intracellulare*, *MGO M. gordonae*. *DM* diabetes mellitus, *COPD* chronic obstructive pulmonary disease, *AS* Ankylosing

### Comparison analysis between patients of NTM disease and NTM colonization

Besides 87 patients with NTM disease, there were still 67 cases of NTM colonization, which did not meet the full ATS/IDSA NTM disease criteria. To determine the risk factors for NTM disease, we performed comparison analysis between the two group patients. Through Chi-square test analysis, there was no significant difference between patients with NTM disease and NTM colonization regarding age, gender, radiographic characteristic and clinical presentation, except that NTM disease patients presented with more fever (27.6%) (Table 4). Remarkably, of 87 NTM disease patients, 56 (64.4%) patients had TB infection history and 17 patients were HIV-positive, which suggested that previous TB and HIV infection significantly increased the risk for NTM disease (Table 4).

**Table 4:** Comparison analysis of comorbidities and clinical features between patients with NTM disease and NTM colonization

	Confirmed case (*n* = 87)	Not meet ATS/IDSA criteria (*n* = 67)
Male	51 (58.6%)	40 (59.7%)
Female	36 (41.4%)	27 (40.3%)
Age (median)	60 (20–82)	66 (19–87)
Radiographic characteristic		
Cavity	33 (37.9%)	25 (37.3%)
Nodule	33 (37.9%)	28 (41.8%)
Bronchiectasis	34 (39.1%)	26 (38.8%)
Comorbidities		
Previous TB	56 (64.4%)^*^	30 (44.8%)^*^
HIV positive	17 (19.5%)^*^	0^*^
Clinical features		
Cough and Expectoration	68 (78.1%)	50 (74.6%)
Hemoptysis	17 (19.5%)	16 (23.9%)
Chest Congestion	8 (9.2%)	6 (9.0%)
Shortness of breath	9 (10.3%)	3 (4.5%)
Fever	24 (27.6%)^*^	9 (13.4%)^*^

*indicates *p* < 0.05 for comparison between columns by Chi-square test

## Discussion

In our retrospective study, we investigated a total of 1461 culture-positive clinical specimens from respiratory patients. The isolation rate of NTM reached 15%, but most of which did not cause disease. Among 87 NTM disease cases, five kinds of NTM species were detected, four slowly growing mycobacteria (*M. intracellulare*, *M. avium*, *M. kansasii*, *M. gordonae*) and one rapidly growing mycobacterium (*M. abscessus*). *M. intracellulare* was the predominant species, accounting for 70.1% of total disease-causing NTM, followed by 11.5% *M. avium* and 11.5% *M. abscessus*. These data were consistent with Zhang’s report that *M. intracellulare* was the most common NTM species in China [23]. Published case reports and series showed that slender, older women were more susceptible to NTM lung infection, especially postmenopausal women [29]. In our study, we did find NTM infection was associated with age, not gender. Actually, with the increasing life span and more availability of people in geriatric age group, the increase in incidence of NTM lung disease is not surprising.

Cough and expectoration were the main clinical presentation of NTM chronic infection, followed by hemoptysis and chest congestion. These symptoms were very similar to tuberculosis [30, 31]. Even though the radiographic characteristics of NTM infection often overlap with MTB [32], we did find something special. The main radiographic presentation of NTM pulmonary disease was bronchiectasis (39.1%), followed by pulmonary cavity (37.9%) and nodules (37.9%). NTM lung disease and bronchiectasis are inextricably linked [33]. NTM infection is very common in bronchiectasis patients [34]. So far, it is still not clear whether NTM are a cause or consequence of bronchiectasis [33]. For pulmonary cavity, the significant point is that multiple and thin-walled cavities were predominant in our NTM disease patients. As NTM frequently co-infects with MTB, it is hard to distinguish NTM and MTB from the cavity feature. However, the multiple cavities indicated the lung structure was severely destroyed and could be predisposed to NTM infection.

As NTM are environmental bacteria, a substantial number of patients have no known risk factors. However, some underlying host risk factors for NTM infection have been extensively studied, like allogeneic hematopoietic cell transplantation (AHCT) [35–37]. Remarkably, the patients with predisposed lung disease are more susceptible to NTM infection [30, 38]. Through review of medical records, we found that 64% NTM patients suffered from previous MTB, and 34 (39.1%) had bronchiectasis. In our study, though 34 patients had bronchiectasis on radiology, only 17 patients displayed clinical bronchiectasis symptoms. We also observed COPD in 6 (6.9%) NTM disease patients. Besides heart disease and stroke, COPD is within the top three causes of human death in the world, which frequently appears together with TB [39]. It’s reported that *M. intracellulare* lung disease was more likely to be observed among patients with COPD comorbidity [23]. Meanwhile, NTM infection are more often observed in HIV patients [5] and cystic fibrosis (CF) patients [40]. *M. avium* complex is the major causative agent of NTM disease in AIDS or CF patients [5, 40]. Consistently, in our study, *M. avium* was also the most frequently isolated NTM in HIV positive patients.

Interestingly, we did not find any remarkable difference between patients with NTM disease and NTM colonization, with regards to demographic feature, radiographic characteristic and clinical manifestation except fever. This was not surprising, because the 67 patients with NTM colonization were TB patients at the same time, 37 with primary TB and 30 with secondary TB. It is difficult to differentiate the clinical symptoms between NTM diseases and TB. Meanwhile, it’s worth noting that 17 of 87 NTM disease patients was HIV-positive. HIV infection usually impairs host immune system and increases opportunistic infection, which might explain why NTM disease patients presented with more fever in our study. Actually, weak immune system and underlying lung disease are included in the risk factor list for NTM disease [39]. Since they had variable lung destruction, those patients with NTM colonization might meet the NTM lung disease criteria in the future and therefore should have closer follow-up.

Taken together, the significance of NTM lung disease has been brought to the forefront. Investigation and analysis of local prevalence of NTM would be worthwhile. Multicentric study is urgently needed to research the epidemiology and other risk factors responsible for NTM infection.

## Conclusions

This retrospective study showed that the most common pathogenic NTM species in Nanjing district was *M. intracellulare*. NTM pulmonary disease was associated with age, instead of gender. The most frequent radiographic presentation of NTM lung disease was bronchiectasis. Previous TB and HIV infection immensely enhanced risk of NTM disease.

## Data Availability

Data relating to this study are contained and presented in this document. Other materials are available from the corresponding author on reasonable request.

## Abbreviations

AHCT: Allogeneic hematopoietic cell transplantation
AIDS: Acquired Immune Deficiency Syndrome
AS: Ankylosing
ATS/IDSA: American Thoracic Society and Infectious Disease Society of America
CDC: Centers for Disease Control
CF: Cystic fibrosis
COPD: Chronic obstructive pulmonary disease
DM: Diabetes mellitus
HIV: Human immunodeficiency virus
MTB: *Mycobacterium tuberculosis*
NTM: nontuberculous mycobacteria
TB: Tuberculosis
WHO: World health organization
